# Causality-driven feature representation for connectivity prediction

**DOI:** 10.3389/frai.2025.1686750

**Published:** 2026-01-15

**Authors:** Bruno Souza, Manuel Castro, Ahmed Esmin, Leonardo Machado, Alexandre Ferreira, Anderson Rocha

**Affiliations:** 1 Artificial Intelligence Lab., Recod.ai, Institute of Computing, University of Campinas, Campinas, Brazil; 2 Department of Computer Science, Federal University of Lavras (UFLA), Lavras, Brazil; 3 Shell, Rio de Janeiro, Brazil

**Keywords:** causal feature learning, connectivity estimation, inter-well interactions, oil field, injector-producer connectivity, causal reasoning, causal theory, dynamic systems

## Abstract

Causal reasoning is essential for understanding relationships and guiding decision-making in different applications, as it allows for the identification of cause-and-effect relationships between variables. By uncovering the underlying process that drives these relationships, causal reasoning enables more accurate predictions, controlled interventions, and the ability to distinguish genuine causal effects from mere correlations in complex systems. In oil field management, where interactions between injector and producer wells are inherently dynamic, it is vital to uncover causal connections to optimize recovery and minimize waste. Since controlled experiments are impractical in this setting, we must rely solely on observed data. In this paper, we develop an innovative causality-inspired framework that leverages domain expertise for causal feature learning for robust connectivity estimation. We address the challenge posed by confounding factors, latency in system responses, and the complexity of inter-well interactions that complicate causal analysis. First, we frame the problem through a causal lens and propose a novel framework that generates pairwise features driven by causal theory. This method captures meaningful representations of relationships within the oil field system. By constructing independent pairwise feature representations, our method implicitly accounts for confounder signal and enhances the reliability of connectivity estimation. Furthermore, our approach requires only limited context data to train machine learning models that estimate the connectivity probability between injectors and producers. We first validate our methodology through experiments on synthetic and semi-synthetic datasets, ensuring its robustness across varied scenarios. We then apply it to the complex Brazilian Pre-Salt oil fields using public synthetic and real-world data. Our results show that the proposed method effectively identifies injector-producer connectivity while maintaining rapid training times. This enables scalability and provides an interpretable approach for complex dynamic systems through causal theory. While previous projects have employed causal methods in the oil field context, to the best of our knowledge, this is the first time to systematically formulate the problem using causal reasoning that explicitly accounts for relevant confounders and develops an approach that effectively addresses these challenges and facilitates the discovery of interwell connections within an oil field.

## 1 Introduction

Causal reasoning is central to how humans understand and act upon the world, particularly in domains such as healthcare (Bica et al., 2020; Bica and van der Schaar, 2022), education (Forney and Mueller, 2022), and public policy (Frumento et al., 2012). Understanding temporal relationships among variables enables stakeholders to answer causal questions, design interventions, and evaluate their impact.

While randomized controlled trials (RCTs) remain the gold standard for causal inference (Pearl, 2009; Bareinboim et al., 2022), they are often costly, time-consuming, or impractical. As a result, there is growing interest in estimating causal effects from observational data.

This is particularly true in oil field operations, where we cannot manipulate injection parameters at will. Our innovative contribution lies in developing a causality-inspired approach that overcomes these experimental constraints through domain-informed feature representations, enabling robust connectivity estimation from purely observational data.

Although Machine Learning (ML) has shown success in various applications, it primarily captures correlations, which can lead to spurious conclusions in structural causal contexts (Ntoutsi et al., 2020). In contrast, some causal methods in order to estimate unbiased cause-and-effect, aim to uncover the underlying generative process, often represented as a causal diagram (Pearl, 2009), offering interpretable, actionable insights of the system. This is especially valuable in settings where understanding variable interactions is as critical as predicting outcomes.

Causal discovery methods for observational data have garnered significant attention for this endeavor in recent years (Spirtes and Zhang, 2016; Runge et al., 2019a) as they can infer an equivalent class of the causal diagram through conditional class representation (Zhang, 2008), which can be further used to estimate interventional queries (Jaber et al., 2022). Alongside traditional approaches like score-based methods (Chickering, 2002), conditional independence (Spirtes and Glymour, 1991), and Granger causality (Granger, 1969), other techniques, including the modern use of deep learning-based models, have been further developed in the field (Liu et al., 2023; Lippe et al., 2022).

These developments are particularly important for time series data (Hernán and Robins, 2010; Bica et al., 2020; Hasan et al., 2023), given that time series formats encompass the majority of scientific and operational data. Given its particularity, specialized algorithms have been developed for temporal data. Notable examples include time-aware adaptations of the PC (Spirtes and Glymour, 1991) and FCI (Entner and Hoyer, 2010) algorithms, such as PCMCI (Runge et al., 2019b) and DYNOTEARS (Pamfil et al., 2020). These methods perform well in dynamic settings, assuming key conditions like acyclicity and causal sufficiency (Runge et al., 2019b) are met.

In practice, the oil field is an example of a complex time-series system where the interactions between injectors and producers evolve over time. Investigating causal links and the interventional effect of an injector on a producer in an oil field represents a significant part of the research effort in the area. Understanding these temporal dynamics is essential for effective reservoir management. If the injector's fluid is not properly managed or there is a lack of clarity regarding the field's interwell connections, it could lead to unintended consequences that undermine desired outcomes. This not only decreases oil recovery but also increases operational costs, as companies must contend with excessive water or gas production and separation issues. Conversely, when operators have a clear understanding of how injected fluids flow underground, through detailed knowledge of connectivity (i.e., causal discovery), and how they impact production (i.e., estimate the treatment effect), they can optimize recovery efforts, minimize waste, and prolong the productive lifespan of the field. Efficient water and gas management, enhanced oil recovery (EOR) factor, and precise reservoir modeling are all contingent upon the fundamental process of accurately understanding and discovering the interwell connectivity across the reservoir.

Unlike traditional causal discovery methods that rely on independence testing or linear models (Jaber et al., 2019a; Pearl, 2009), our framework adopts a pragmatic causal feature learning approach inspired by domain expertise (Kumar et al., 2018, 2020). Instead of seeking formal identifiability, we focus on uncovering physically consistent causal patterns that reflect real-world approaches for estimating the likelihood of connectivity between wells. In an oil field, connectivity estimation is an open challenge, as the available data often presents complex, detailed information that represent the underlying generative processes that drive those relationships. This challenge is further extended by potential hidden confounding factors of the reservoir. As a result, uncovering these causal connections becomes significantly more difficult. Natural reservoir pressure variations, geological heterogeneity, and interactions between multiple wells introduce complexity, while manual control adjustments must further alter the system's dynamics (see Section 3). To further complicate, the oil field operators often cannot directly manipulate injection parameters at will and observe isolated effects, making it difficult to disentangle causal relationships (Pearl, 2009) (i.e., determining whether a causal relationship exists and whether the effects observed can be attributed to specific interventions rather than confounding variables). Additionally, system responses frequently exhibit time delays, adding another layer of complexity for experimental data acquisition. Instead, one alternative is to rely on observational data from tracer (chemical compounds) tests and pressure monitoring that aims to seek mutual variation in the signal that corresponds to the causal connection between wells. Thus, our proposed framework aims to capture domain-consistent causal patterns modeling causal dependencies.

In recent years, Causal Feature Learning (CFL) has emerged as an alternative tool to uncover and explain relationships in complex systems (Chalupka et al., 2016; Hannart and Naveau, 2018). CFL is a causal reasoning framework rooted in the language of causal graphical models aimed at discovering causal relations from high-level variables (i.e., aggregated features that capture broad patterns) from low-level data (i.e., the possible observational measurements) and at reducing the experimental effort to understand confounding among the high-level variables. The CFL aims to identify features that present the necessary information for direct causal detection of an outcome. For example, some studies (Chalupka et al., 2017) leverage CFL to construct macrovariables that preserve the underlying causal relationships between the microvariables. For instance, rather than tracking the kinetic energy of every particle, we can monitor the room's temperature, which encapsulates the essential information. We innovate within this framework by developing domain-specific causal features that translate reservoir engineering expertise into measurable connectivity signatures.

In the context of reservoir, the application of causal feature learning is particularly interesting, as it could represent the causal information translating the way experts assess well connectivity by examining lagged mutual variations between injector and producer curves with the primary challenges lying in the confounding interactions between wells, along with lagged influences and the inherent complexity of the system's behavior. In low-level data, the complexities of the system can obscure the true connectivity and effect of injection strategies on production levels. Additionally, the absence of controlled experimental interventions further complicates the analysis. Therefore, by leveraging the higher-level feature learning approaches, that preserves and allows quantification of the degree of co-variation or responsiveness between wells is of extreme interest in the field. With the causal signatures of connectivity embedded in the data, providing a statistical approximation of the underlying physical causality, researchers can gain valuable insights and develop AI-based models that preserve the inferred causal information for connectivity estimation between wells, facilitating more informed decision-making and enhancing understanding in reservoir management.

We emphasize that our approach does not claim formal causal identifiability in the strict sense of Pearl's do-calculus or potential outcomes framework (Pearl, 2009; Hernán and Robins, 2010). Rather, we develop features inspired by causal principles that capture domain-consistent dependencies indicative of underlying physical connectivity. Traditional independence-based causal discovery methods empirically proved not to be well-suited for this environment, where causal influence appears as subtle, lagged covariations between injector and producer signals mediated by a partially observable reservoir. Our causal-inspired feature learning approach leverages these concepts as proxies for causal information, offering a more practical, interpretable, and empirically robust alternative.

### 1.1 Contribution

Throughout this paper, we focus on structural causal discovery, estimating the causal connection of injector–producer relationships. We approach the oil field formulation problem through a causal lens, offering a structured formulation of the key challenges that arise when attempting to infer interwell connectivity and causal influence within the oil field. To address this complex problem, we were inspired by the experts' approach and leveraging the CFL to capture meaningful representations that circumvent the unobserved factors affecting connectivity. Our method constructs independent pairwise feature representations that implicitly encode the influence of confounders while ensuring that external phenomena do not distort the inferred relationships. By learning these balanced comparative representations of injector-producer pairs, our approach aims at mitigating biases and enhancing the reliability of connectivity inference. Additionally, pairwise connectivity inference allows us to work with permutation invariance analysis, which is crucial for statistical efficiency in structural learning and facilitates generalization to larger problem instances than seen during training. We leverage the tracer data (i.e., a chemical substance added to the fluid to monitor and identify connectivity between wells), which is a scarce yet valuable resource, to train machine learning models that estimate the probability of connectivity between injection and production wells.

To validate our approach, we first conduct experiments on synthetic and semi-synthetic datasets, ensuring that our model generalizes across controlled and partially real-world scenarios. We then extend our study to the Brazilian Pre-Salt field, a highly challenging and geologically complex environment, using both public synthetic (from simulations) and private real-world data. Our results demonstrate that the proposed method effectively identifies injector-producer connectivity while maintaining computational efficiency, achieving training times of under one minute in nearly all tested cases. By integrating causal reasoning principles with ML, our approach enhances decision-making in reservoir management, offering a scalable and interpretable solution for complex subsurface flow modeling.

## 2 Related work

### 2.1 Time series causal discovery methods

The most widely adopted notion of causality in computer science is given by the Structural Causal Model (SCM), introduced in the early 20th century and now championed by Judea Pearl (Pearl, 2009). The field of causal discovery focuses on identifying and modeling the causal relationships between variables using observational data. It aims to recover a Directed Acyclic Graph (DAG), which visually represents these relationships, by analyzing patterns and dependencies within the data.

Numerous efforts have been made to address the challenge of establishing causality within time series data (Hernán and Robins, 2010). These methods can be broadly categorized as follows:

#### 2.1.1 Methods based on granger causality

One of the earliest approaches to causality in time series is Granger causality (Siggiridou and Kugiumtzis, 2015). It states that a time series *X* *Granger-causes* *Y* if past values of *X* improve the prediction of *Y* beyond what *Y*'s own history can provide. This is commonly modeled using a Vector Autoregressive (VAR) framework:


$$\begin{array}{l} Y_{t}=\overset{\tau_{\text{max}}}{\underset{\tau =1}{\sum }}a_{\tau }Y_{t-\tau }+\overset{\tau_{\text{max}}}{\underset{\tau =1}{\sum }}b_{\tau }X_{t-\tau }+\eta_{t} \end{array}$$ (1)

Here, *Y*_*t*_ is influenced by its own lags (via *a*_τ_) and potentially by *X* (via *b*_τ_). If any *b*_τ_ ≠ 0, *X* is said to Granger-cause *Y*. The noise term η_*t*_ accounts for unobserved influences.

Granger-based methods rely on key assumptions (i) *Stationarity:* Time series should be stationary, (ii) *Causal Sufficiency:* All relevant variables must be observed, and (iii) *Temporal Order:* Causes precede effects. A main limitation is the assumption of linearity. However, extensions to multivariate and non-linear settings have been proposed (Haufe et al., 2010; Siggiridou and Kugiumtzis, 2015).

#### 2.1.2 Conditional independence-based methods

Conditional independence-based methods offer a principled approach to uncovering causal relationships in time series. These methods test whether a variable *X*_*k*_ at time *t*−τ is independent of another variable *X*_*l*_ at time *t*, given the past of both, capturing complex temporal dependencies in dynamic systems. Their validity rests on key assumptions: (i) causal sufficiency and (ii) time-order. In addition, variables should be conditionally independent given their parents if not directly connected (Spirtes et al., 2000, Chapter 3).

Recent studies (Runge et al., 2019b; Jaber et al., 2019b; Jeong et al., 2025) show these methods' effectiveness across domains. However, in complex settings like oil fields, where latent confounders and high dimensionality are common, these approaches face limitations. Specifically, constraint-based methods may require exponentially many tests (Pearl, 2009), and violations of causal sufficiency can distort time-order inference, affecting both accuracy and scalability in practice.

#### 2.1.3 Deep learning-based models

Recent approaches have leveraged neural network architectures, including Multi-Layer Perceptrons (MLPs), Recurrent Neural Networks (RNNs), and Convolutional Neural Networks (CNNs) for causal discovery and inference estimation (Nauta et al., 2019). DYNOTEARS (Pamfil et al., 2020) is a score-based method that jointly estimates contemporaneous and time-lagged dependencies by minimizing a penalized loss under an acyclicity constraint. Similarly, NTS-NOTEARS (Sun et al., 2021) extends this framework to nonlinear time series using 1D CNNs to model parent-child dependencies in dynamic Bayesian networks. It also enforces acyclicity via continuous optimization.

Deep Learning (DL) models have been widely used in healthcare for causal inference and counterfactual estimation due to their ability to model complex nonlinear relationships (Bica et al., 2020; Bica and van der Schaar, 2022). Feuerriegel et al. (2024) highlight the advantages of causal ML over traditional approaches and outline key implementation steps, recommending its reliable use. Nauta et al. (2019) review causal discovery methods for time series, noting their limitations: sensitivity to hidden confounders and reliance on stationary data, which can lead to unreliable results when violated. When applied to non-stationary data, these methods may produce unreliable results, potentially leading to misleading conclusions about causal relationships.

### 2.2 Causal artificial intelligence

Causality is driving the next wave of advancements in Artificial Intelligence (AI) (Glymour et al., 2014). By integrating causal logic into ML paradigms, researchers aim to enhance human-like reasoning capabilities and promote the development of emerging areas such as representation learning (Schölkopf et al., 2021), reinforcement learning (Bareinboim et al., 2021), and large language models (Vashishtha et al., 2023). Incorporating causal assumptions allows researchers to utilize observational data to tackle “what if” questions, thereby inferring potential interventional or counterfactual outcomes that are unobserved.

In complex problems, practitioners often lack access to the true DAG that defines the causal relationships between variables. The field of causal discovery endeavors to reconstruct a causal diagram from the available data. Unfortunately, it is widely acknowledged that uniquely identifying the true causal diagram from observational data (i.e., especially when experiments are prohibitive) is generally unfeasible without adhering to assumptions (Peters et al., 2013).

To address these complexities, a branch of causal AI named CFL seeks to combine the strengths of feature learning with causal reasoning. CFL is an emerging area within DL that holds substantial promise but is still in its formative stages (Chalupka et al., 2017, 2016; Hannart and Naveau, 2018). While many cutting-edge DL techniques excel at generating geometric representations of modeled entities, they often struggle to capture meaningful representations from a causal perspective. The goal of CFL is to establish theoretical frameworks and learning algorithms that are accurate but also robust, generalizable, and fair.

In general, CFL constructs macro variables that preserve the causal relationships between variables. These macro variables are intended to reduce the complexity of finding causal relationships in data by identifying a small number of relevant macrostates that can be used to test causal hypotheses. In other words, the task of CFL aims to aggregate the information into a more abstract, high-level representation involving fewer variables and relations that should be easier for experts to reason about. This must be done so that the act of creating a representative feature does not affect the causal relations among variables. For instance, rather than trying to monitor the kinetic energy of every individual particle in a room, we can simply observe the room's temperature.

Practical applications of this integration can be seen across various ML domains, including supervised learning (Kyono et al., 2020), missing data imputation (Kyono et al., 2021), domain generalization (Bica et al., 2021), and fairness (Van Breugel et al., 2021). In real-world problems, domain expertise often provides valuable inductive biases that can guide causal representation learning (Chalupka et al., 2016). In this sense, expert-guided feature construction can act as a bridge between purely data-driven learning and physically interpretable causal reasoning. In our work, the causal features were deliberate and motivated by domain expert approaches. In reservoir engineering practice, well connectivity is typically inferred through analyses of lagged mutual variations, pressure interferences, and dynamic responses between wells. Accordingly, our features were designed to capture these meaningful relationships, ensuring that the resulting model aligns with how experts interpret injector–producer interactions, while remaining consistent with causal feature learning principles.

In our research, we aim to leverage representation features (i.e., construct macro variables that implicitly retain connectivity information while reducing dimensionality) to tackle the challenges of causal discovery in time series data, especially within the oil field.

### 2.3 Connectivity discovery in oil field

In recent years, the challenge of uncovering causal relationships in time-series data has garnered considerable attention (Runge et al., 2019b). Traditional causal discovery methods, such as Granger causality or the constraint-based approach, often rely on statistical assumptions like stationarity or sufficiency, which may not hold in complex, dynamic systems. These methods can struggle to reveal accurate causal structures in complex systems with significant non-linearities and temporal dependencies.

The Capacitance-Resistance Model (CRM) (Sayarpour et al., 2009) method tries to discover connectivity by an analytical technique used to visualize and assess the relationships and interactions between different components in a system, such as injectors and producers in an oil field (Wang et al., 2019). By modeling these connections, CRM aims to identify causal relationships that explain how changes in one variable (e.g., injector operations) affect another variable (e.g., production outcomes). However, it can fail due to low-quality data, hidden confounding factors, or incorrect assumptions about the system dynamics, leading to misleading conclusions.

In response to traditional causal discovery and CRM methods, Castro et al. (2023)'s work introduces the Aleph model. This novel approach leverages the power of ensemble models to uncover causal relationships in time-series data in the context of oil field production. Instead of relying on strict assumptions about the data, Aleph focuses on the importance of time features—variables that directly influence the prediction outcomes of interest.

At the heart of this approach is feature importance, quantifying how much a particular feature contributes to the model's predictive power. In ensemble methods, feature importance is calculated by evaluating how much each feature reduces the error or uncertainty in the model's predictions. Their methodology employs an iterative approach to enhance forecast models, which is a concept closely related to Granger Causality, and establishes causal connections based on the observed improvements in predictive performance.

However, a notable limitation of the Aleph model is its failure to account for complexity and the latent dynamics inherent in oil fields, which are critical elements in real-world scenarios. This shortcoming generally leads to a dilemma with limited applications of Granger causality, mostly to bivariate analyses that cannot account for indirect links or common drivers (Runge et al., 2019b). Furthermore, the model's performance is influenced by its order-dependent nature, wherein the results can vary significantly depending on the sequence in which potential drivers are introduced. This introduces inconsistency in establishing connectivity, particularly in dynamic real-world situations. Lastly, the model faces scalability challenges, as its effectiveness diminishes with larger datasets or more extensive oil fields, reducing its applicability and practical utility in broader contexts.

This work demonstrates that even without traditional causal algorithms, utilizing feature representation along with ML offers a compelling way to uncover insights into complex systems. It represents a pioneering step in using advanced machine-learning techniques to discover connectivity with causal reasoning about the process solely from production data.

Therefore, our framework focuses on the structural discovery stage, estimating the probability of connectivity between wells. Specifically, at this point, our method employs causal discovery, leveraging causal features to establish the relationships between injector and producer wells. We strongly believe that tackling this problem first is fundamental, as it provides the underlying connection upon which future causal inference methods can operate.

## 3 Problem formulation

In this section, we present the problem formulation for causal discovery in time series. One of our key contributions is the application of a connectivity framework to the oil field problem through a causal lens. We define the graph structure, highlight the main challenges in inferring connectivity, and outline assumptions that may fail in real settings.

### 3.1 Time series causality

Causal discovery in time series seeks to uncover relationships between variables over time. Given a dataset $X=\left\{X_{1},\ldots ,X_{N}\right\}\in {\mathbb{R}}^{N\times T}$, the goal is to identify causal links and their corresponding time lags.

Two major challenges arise: (a) high dimensionality, often involving many variables, and (b) strong interdependencies. Correlations reflect not only causal effects but also autocorrelation, indirect paths, and hidden factors.

Standard assumptions include *time ordering, causal sufficiency*, and *conditional independence* with faithfulness (Assaad et al., 2022; Pearl, 2009). Under these assumptions and a DAG, one may answer causal queries. However, hidden confounders—unobserved variables affecting both causes and effects—introduce bias and limit the applicability of these methods in practice.

Time-varying hidden confounders further complicate inference (Mansournia et al., 2017), as their evolution can induce spurious temporal effects. If unaddressed, such confounding biases affect estimates.

**Figure 1:**
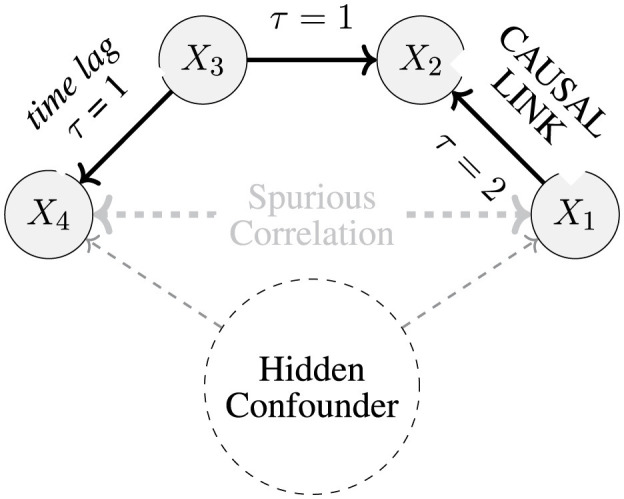
In this general example, *X*_2_ acts as a collider, blocking the causal path between *X*_1_ and *X*_4_. However, the hidden confounder introduces spurious correlations that can adversely affect causal analysis. If the system does not control for this confounder or does not adopt a suitable causal approach to manage it, the model is likely to perform poorly and identify suboptimal correlations.

The goal of causal discovery is to estimate a sparse causal network. Figure 1 illustrates a system with four observed variables and a hidden confounder. The task is to recover true dependencies, including nonlinear relationships and time lags, while avoiding spurious associations created by unobserved factors—even when colliders (e.g., *X*_2_) are present.

Hidden confounders violate causal sufficiency by inducing artificial associations and can disrupt time ordering. They also violate the Causal Markov condition, creating false conditional independencies, for instance, variables appearing independent despite being linked through the confounder.

### 3.2 Oil field problem formulation through causal lens

The oil field system exemplifies a complex, dynamic system, with probably linear and non-linear interactions and hidden variables. In such systems, various observed and hidden variables, such as pressure, temperature, flow rates, and production levels, interact in intricate ways, influenced by numerous underlying mechanisms. Specifically, it is challenging to obtain all the relevant variables that contribute to the functioning of the system (i.e., often due to the lack of knowledge about the true system). Consequently, we argue that the available variables in oil field data are typically insufficient to capture the level of detail required to answer specific causal questions effectively. This discrepancy can impede traditional causal algorithms and complicate their ability to capture the complexities and nuances of the underlying processes accurately.

One of the primary challenges is the presence of hidden and frequently time-varying information. When uncontrolled, these unmeasured factors induce spurious correlations that obscure true causal relationships. For example, regulatory changes, geological heterogeneity, or operational actions can simultaneously affect multiple observed variables, complicating causal interpretation. Moreover, the system is not isolated: operator decisions and external factors may introduce additional sources of variability that interact with reservoir dynamics. These interacting processes can create cyclic dependencies, shift temporal patterns, and temporarily mask true injector–producer connectivity.

Another significant aspect to consider is the frequent occurrence of feedback loops, where the effect of one variable can cycle back to influence other variables within the system. Additionally, time-varying confounding, which can arise from past exposures, complicates the situation further, especially when feedback exists between exposure and outcome. In some cases, the challenges posed by feedback loops are addressed by utilizing causal time series graphs, which incorporate the time-order assumption prevalent in natural complex systems, provided there are no confounding variables present. Typically, these time series approaches discretize time, assuming that measurements possess sufficient temporal resolution to prevent interactions between variables during the same discrete time events. This effectively means that instantaneous effects between variables are often excluded from the analysis, which can simplify modeling but may also overlook critical dynamics (Runge et al., 2019b).

That way, we encounter three significant challenges in the context of oil field operations. Even with arbitrarily high temporal sampling, the low-level signals recorded in operational datasets are often difficult to interpret in causal terms. Injection and production traces are smoothed, delayed and superposed by subsurface transport and reservoir dynamics, so the measurable time-series rarely expose clean, localized perturbations that uniquely identify well-to-well influence. Consequently, models can struggle to extract the causal information for connectivity, i.e., frequent sampling alone does not guarantee the underlying physical causal fingerprints we aim to detect. Second, operators' involvement introduces feedback loops that can be difficult to predict. For example, fluctuations in production rates can impact pressure dynamics, subsequently affecting future production capabilities. Operators who can actively alter the system's dynamics (e.g., closing the choke or altering the fluid injection, which in turn affects this complexity, making it essential to account for these unpredictable influences in any analytical framework. Third, even when data is abundant, the variables typically available in oil field datasets are often insufficient to capture the true level of detail needed to answer specific causal questions.

**Figure 2:**
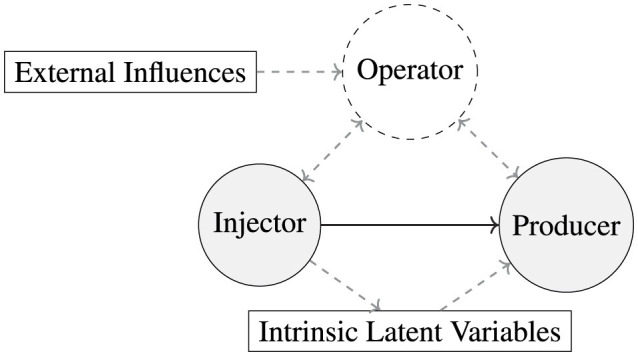
Proposed DAG representing the causal relationships within the oil field system. The operator node has the potential to introduce loops in the system, which often go unnoticed. Additionally, intrinsic variables can significantly alter the entire system, negatively impacting many causal discovery algorithms. For example, scenarios such as a closed choke or geographical obstacles can temporarily disrupt connections. These factors must be considered to ensure accurate causal analysis and model performance in the context of oil field.

Figure 2 illustrates our proposed DAG formulation of the oil field system to identify and expose the potentially time-varying hidden confounder, hidden intrinsic phenomena, and their possible relations. In addition, Table 1 summarizes the causal formulation of the oil system through the causal lens, highlighting the aspect to account for and its descriptions.

**Table 1:** Causal formulation problem of the oil field system.

**Aspect**	**Description**
Hidden confounders	Unobserved geological and operational factors jointly influence multiple measurements, creating spurious dependencies that obscure true connectivity.
Internal and external influences	Operator decisions and external interventions introduce latent shifts in system behavior that can mask or distort the statistical signatures needed for causal inference.
Feedback loops	Interactions such as production affecting pressure—and pressure guiding future operator actions—induce feedback structures that complicate standard causal modeling.
Data quality	Even with high-frequency sampling, low-level signals are mixed and smoothed by subsurface processes, limiting their causal interpretability for identifying connectivity.
Dynamic systems	Time-varying reservoir behavior, shifting well roles, and operator adjustments alter dependency patterns over time, introducing additional challenges for stable causal analysis.

In summary, a deep understanding of the system's dynamic behavior is essential for meaningful analysis. By framing the problem using causal reasoning about the process, we can better represent plausible generative processes and reduce systematic biases. Our approach approximates causal dependencies that are consistent with reservoir physics, operational mechanisms, and domain knowledge. The comparative causal features we employ capture statistical dependencies that are informed by causal reasoning, enabling the model to learn connectivity patterns even when strict causal identifiability is not achievable. Causal principles thus strengthen the interpretability and reliability of the proposed framework, supporting more informed operational decisions despite the inherent complexity of the oil field system.

### 3.3 Oil field connectivity fundamental objectives

Determining the connectivity between injectors and producers remains an open challenge that precedes the application of causal inference algorithms. In practice, the physical structure of the reservoir is only partially observed, and connectivity itself represents a challenge that must first be estimated before any reliable interventional or counterfactual analysis can be performed.

In addressing the intricate challenges present in understanding the connectivity between injectors and producers, our primary goal is to mitigate the dynamicity of the system and effectively “minimize” the reservoir influences that obscure direct causal relationships. This entails creating a representation that intrinsically maintains the necessary information and addresses the complexities arising from the system. Subsequently, we frame the problem as a classification task, classifying the connections into positive or negative classes based on their features. This perspective allows us to systematically categorize the interactions between the injector and producer variables while leveraging the context information (e.g., tracers). In addition, we adopt the assumption of independence between each pairwise analysis (i.e., each injector-producer pair under study) conducted throughout our methodology.

This assumption emphasizes that the response curve for each unit (i.e., connected pair) is never related to the interactions between other units. This independence assumption is respected as we always independently analyze the pairwise connectivity. It simplifies our approach and potentially overcomes the limitation of Castro et al.'s (2023) work related to the ordering. In addition, by treating each analysis in isolation, we can minimize the potential biases introduced by intertwining variables and create clearer insights into the causal structure within the oil field system.

Through these strategic objectives, we establish a solid foundation for our causal analysis: mitigating dynamicity, framing the issue as a classification task, ensuring independence between wells, and emphasizing a data-driven approach by formulating feature learning from causal reasoning.

## 4 Methods

To address the causal connectivity challenges formulated in Section 3, we propose a framework grounded in causal feature learning. Our framework follows a structured pipeline for connectivity estimation grounded in causal feature learning principles. The core insight is that comparative pairwise features, informed by domain expertise and causal reasoning, can capture essential connectivity signatures while remaining robust to dynamic reservoir attributes. Our methodology is structured around three key questions: (i) What rationale supports our causal feature representation? (ii) How does our framework overcome the identified oil field challenges? (iii) What implementation steps ensure practical applicability?

### 4.1 Method's rationale

Our methodology is motivated by the need to reduce biases introduced by time-varying hidden variables and by operator-driven interventions that affect reservoir dynamics. To address these issues, we construct a static comparative feature representation, inspired by CFL theory, that captures pairwise interactions between producers and injectors. In total we create six features, refer to Section 4.3, that were selected to capture a comprehensive set of statistical dependencies that reservoir engineers use to assess connectivity, including linear and non-linear relationships, time-lagged influences, and frequency-domain synchrony. This design ensures permutation invariance, improving statistical efficiency for structural learning. Combined with a classifier, the framework learns to map these features to the estimated probability of injector–producer connectivity.

The underlying rationale is that comparative pairwise features, grounded in causal reasoning and domain expertise, encapsulate essential information for uncovering injector–producer dependencies while remaining robust to dynamic and latent reservoir attributes. This idea is rooted in the notion that pairwise comparative representations allow the model to extract the key relational patterns required to infer connectivity.

By developing high-level abstractions guided by causal reasoning and domain expertise, we aim to overcome the limitations imposed by the low-granularity nature of the observed data, which often hides meaningful relationships behind operational noise and unobserved confounders. In practice, these abstractions mitigate challenges caused by the limited detail in the raw signals, where important interactions are obscured by noise, unmeasured controls, and latent geological factors.

**Figure 3:**
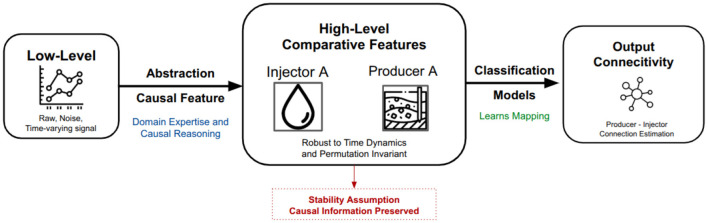
This diagram outlines our approach for estimating injector–producer connectivity. It transforms noisy low-level data into static comparative features using causal reasoning to mitigate biases from complex interactions and time-varying occurrences. This stable representation is fed to a classifier for a scalable, bias-reduced estimation of connectivity probability, bypassing the need for unstable dynamic modeling.

Figure 3 illustrates the method's rationale; therefore, because the available data provide a lower level of abstraction than what is needed to meaningfully characterize reservoir complexity, we leverage comparative domain knowledge to derive stable high-level features.

This static representation preserves the information necessary to account for the time-varying confounding factors that typically bias dynamic analyses, while avoiding the instability of explicitly modeling full reservoir dynamics. Thus, instead of relying on dynamic modeling—often unstable or data-hungry in real-field settings—the static representation retains the essential structure needed for robust inference.

These features are designed to reflect statistically meaningful dependencies without altering the underlying causal structure of the system, ensuring that the act of creating representative features does not introduce spurious relations or distort causal effects. This avoids introducing artificial dependencies while reducing noise from transient dynamics and operator decisions.

By grounding our analysis in this stable comparative representation, the framework offers a scalable and interpretable approach to estimate injector–producer connectivity without requiring explicit dynamic modeling, which is particularly valuable for real-field applications. Ultimately, this static comparative structure enables the model to learn reliable connectivity probabilities while sidestepping the need to model full reservoir dynamics explicitly.

#### 4.1.1 Underlying assumptions and limitations

We acknowledge the presence of unobserved geological and operational factors that may influence both injector and producer behavior. Our framework treats these as latent information that are assumed to act in a relatively monotonic and stable manner during the observation period, allowing the causal features to remain informative of the true connectivity. This assumption is supported by the physical consistency of reservoir dynamics, where geological properties evolve slowly compared to operational adjustments, ensuring that causal patterns derived from comparative features remain valid.

In addition, it is important to note that, due to the current lack of detailed geological data, i.e., limiting the geological validation, our evaluation relies on tracer-confirmed connections to ensure methodological reliability, representing the conservative labeling strategy. The uncertainty in the model's predictive capacity reflects both data limitations and the inherent uncertainty in estimating connection strength. Furthermore, the implicit definition of high or low connectivity, whether as a probabilistic distribution or under alternative modeling frameworks, remains an open direction for future research.

#### 4.1.2 Modeling and data assumptions

Our framework relies on several core assumptions to ensure theoretical consistency and reproducibility. First, each well's production and injection signals are assumed to be locally stationary within the defined analysis windows, which can be manually selected to exclude periods of extreme fluctuation or prolonged shutdown. In our case, we applied normalization to mitigate residual non-stationary effects. Second, we assume a consistent sampling frequency across wells, a realistic condition in industrial operations, where data acquisition is standardized. Occasional missing values were interpolated within stable windows to preserve temporal alignment. Finally, we acknowledge the existence of latent geological and operational confounders that may influence both injectors and producers' rates. However, these latent factors are assumed to act in a relatively monotonic and stable manner during the observation period, allowing the derived causal features to remain informative of the true connectivity, which stems from the natural covariation dynamics within the reservoir.

### 4.2 Proposed framework

As aforementioned, our approach transforms noisy low-level time series data into stable static representations. The proposed framework achieves this through three key stages: (1) pairwise candidate generation and filtering using causal discovery algorithms, (2) extraction of domain-informed causal features that capture statistical dependencies indicative of physical connectivity, and (3) supervised classification using limited tracer data to estimate connection probabilities.

**Figure 4:**
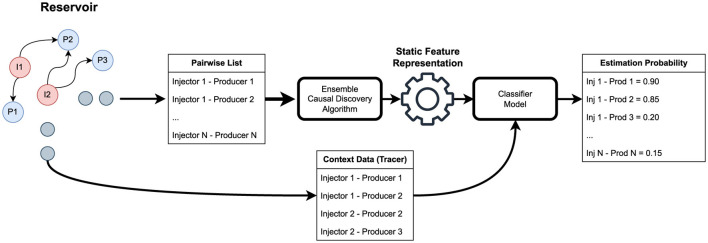
Our framework for estimating connectivity leverages causal reasoning through a structured approach. We begin by creating a mutually independent pairwise list of all injector-producer combinations. Next, we apply causal discovery methods to refine this list, retaining only the viable pairs. With these filtered pairs, we develop our pairwise static feature representation that will be used to train our classifier model. Finally, using limited contextual data (i.e., the tracers), we train our model to predict the potential connectivity between producers and injectors.

Figure 4 outlines a systematic methodology designed to estimate the connectivity between injectors and producers within an oil reservoir while overcoming confounding variables and the system's variability. The process begins with identifying relevant components and progresses through several essential stages, each contributing to our understanding of the system's dynamics.

The starting point is the reservoir, with injectors and producers. The arrows indicate the connections. The daily field's operation sets the context for our analysis (i.e., the scenario). From the reservoir, we create a list of all possible connections, along with the variables corresponding to the fluid injected by the injector. From the producer, we record the production rate and the pressure on the choke.

In traditional constraint-based causal discovery methods, causal relations are inferred through conditional independence tests. However, this approach is not well-suited to oil field data, where each well (injector or producer) is represented by a multivariate time series connected through an unobserved physical reservoir. In practice, we empirically verify that a well-established causal discovery algorithm, although not performing well in finding connectivity, is useful in removing unlikely connectivity, which is demonstrated to be helpful as a two-step filtering connectivity discovery.

Therefore, our approach employs PCMCI (Runge et al., 2019b) or/and DYNOTEARS (Pamfil et al., 2020) algorithms to identify potential connections. If these methods confirm a connection, we consider it a candidate for further evaluation in the subsequent step.

Next, we transition to what is probably the most important part of our framework: the construction of statistical feature representations by computing causal-comparative features. In engineering practice, experts assess well connectivity by examining lagged mutual variations between injector and producer curves, interpreting a visible change in a producer's trend as a response to a prior change in a connected injector. Therefore, in this phase, we propose to build a comparative static representation which quantifies the degree of covariation or responsiveness between wells. These features act as proxies for causal signatures of connectivity embedded in the data, providing a statistical approximation of the underlying physical causality. We extract features that summarize the interactions between injectors and producers. This representation is structured to retain vital connectivity information while minimizing the impact of transient variables that may distort our analysis.

Once the static features are established, we implement a classifier model, Random Forest. This model maps the relationships leveraging the generated static feature representation and the scarce context data (i.e., the tracers), helping to identify underlying causal links between the injectors and producers. Here, the classifier outputs the estimated connectivity probabilities for each injector-producer pair. It is designed to estimate the probability of connectivity between injector-producer pairs and allow the operator's analysis to be flexible based on the nature of their interactions. These probabilities provide actionable insights, guiding operators in their decision-making processes.

### 4.3 Implementation details

We implemented a systematic approach emphasizing a data-driven methodology to construct our framework for analyzing the connectivity between injectors and producers in the oil field system. After identifying potential causal links through a preliminary filtering stage, we proceed to extract comparative causal features that encode domain-relevant dynamics, later used for supervised classification. Our approach is designed to create a static feature that intrinsically overcomes complex interactions while mitigating the system's inherent dynamicity. In addition, we apply the scenario concept: a time window is defined, and only injectors and producers active in this time window are analyzed. The scenario is intended to mitigate variations in each well's role and decrease the data missing, for these are often presented in real-world data.

#### 4.3.1 Creating pairwise data-driven features

We initiated the process by developing pairwise data-driven features that focus on mimicking a static representation of the system. The idea is that the pairwise static representation can have information about the connectivity of the injector-producer under analysis, while intrinsically keeping the information created by the hidden confounders. This representation is inspired by the concept of propensity scores (Guo and Fraser, 2014), formulated as :


$$\begin{array}{l} e\left(X\right)=P\left(T=1|X\right) \end{array}$$ (2)

where *X* = *x*^1^, …, *x*^*M*^ is a vector of representation with dimension *M*. Given the observed features, we seek to mitigate bias by estimating the probability of a unit being treated *T* (i.e., connected) given the vector representation. By relying on pairwise interactions, we capture the statistical relation information between each injector and producer pair while maintaining a simplified, static perspective.

For each pair of injectors and producers, we implemented a data-driven representation strategy using the fluid injected, the production rate, and the pressure of the choke as variables. From this approach, we generate six pairwise comparative representations (i.e., vector *X* with dimension six) inspired by the information theory field (Ash, 2012), potentially preserving the causal information. This higher-level variable *X* aim to encapsulate the representation between the injector-producer while keeping the intrinsic dynamic information about the connectivity:

1. **Maximum correlation**: It identifies the strongest linear relationship between the production and the fluid injected.
2. **Granger causality**: Assesses whether injector time series improve the prediction of the producer, indicating a directional influence.
3. **Mutual information**: Measures the amount of information obtained about the producer through the injector, reflecting non-linear dependencies.
4. **Power spectral density correlation**: It analyzes the injector and producer frequency components, providing insights into periodic behavior and synchronization.
5. **Conditional mutual information**: Evaluates the degree of association between injector and producer, conditioned on the pressure of the choke, accounting for potential confounding factors.
6. **Distance**: As prior knowledge of the task, distance is crucial in informing the connectivity estimation.

These six features were selected because they collectively capture the key signatures reservoir engineers use: directional influence (Granger), linear and non-linear dependencies (correlation, mutual information), frequency patterns (spectral density), confounder-adjusted relationships (conditional MI), and spatial constraints (distance).

It is important to note that in our tests, the distance as a feature was only used in the analysis of real-world data. In contrast, for the semi-synthetic and UNISIM-II datasets, we relied solely on maximum correlation, Granger causality, mutual information, and power spectral density and conditional mutual information.

The decision to rely on manually constructed causal features was deliberate and grounded in reservoir engineering practice. Experts typically evaluate well connectivity through lagged mutual variations and pressure interferences, which inspired our feature design. This handcrafted approach ensures that the model aligns with interpretability and domain intuition, while also improving computational efficiency and training stability as demonstrated in Section 5. We also argue that, although representation learning techniques could provide richer predictive features, they would likely overfit under our limited labeled data scenario and reduce interpretability.

#### 4.3.2 Using scenarios to decrease dynamicity

To further address the challenges posed by the system's dynamics imposed specifically by operators, we employed specific scenarios (i.e., stratification of the data in small time windows) aimed at decreasing the variability of the data, as the probability of opening or closing determined well would decrease. By defining and analyzing distinct operational scenarios, we can decouple the effects of transient conditions, enabling clearer insights into the fundamental connectivity relationships. In addition, applying the proposed scenarios also shows to be beneficial for such feature-based classifier techniques. With smaller window sizes, the features capture information more effectively, enhancing their relevance to specific connections.

#### 4.3.3 Leveraging context data and train

Our framework also capitalizes on context data, specifically tracer information, to inform the labeling process for our classification task. In this context, pairs identified with tracer information are labeled as connected. Conversely, in the real-world data, we leverage our prior knowledge about the high probability of non-connection arising from distant pairs to assign them as negative labels. The other pairs are not used to train our model. In the semi-synthetic and UNISIM-II data we have access to the positive and negative connectivity data. This dual-labeling method allows us to train a classification model and try to find the approximate function that leverages the pairwise static representation to estimate the probability of connection.

We also clarify that, due to the limited availability of labeled tracer data, we adopted a conservative labeling strategy, using only high-confidence positive connections confirmed by tracer tests and thoroughly validated negative pairs that are physically implausible based on distance and distinction signal to be connected. While this minimizes label noise, we acknowledge that explicitly modeling label uncertainty could enhance robustness and interpretability.

With the methodology and feature-based classifier defined above, we next evaluate the approach across semi-synthetic, benchmark, and real-world reservoirs. In our training setup, we utilize a random forest classifier trained on a causal pairwise representation, augmented by expert-derived negative labels based on the distances between injector and producer locations, and implement a bootstrapping strategy that involves 100 resampling iterations to mitigate overfitting due to the imbalance between positive and negative samples. Subsequently, we use the trained model to evaluate in the full system the probability of each pair of injector-producer being connected.

## 5 Applications

Having established the conceptual foundation, we now describe how the framework is implemented in practice. In particular, we demonstrate how our causal feature representation, inspired by expert reasoning about injector–producer dynamics, translates into measurable performance gains across both synthetic and real-world settings.

Using our static feature representation, we evaluated our framework across multiple datasets to assess its ability to uncover causal relationships between time series.

First, we tested on a linear semi-synthetic dataset where we simulated the choke, a latent time-varying variable, and the production rate. The fluid-injected variables in this dataset were derived from real-world data, providing a realistic foundation for our simulations. Next, we applied our approach to the UNISIM-II dataset, a synthetic oil production field that offers insights into causality within time series data related to a synthetic reservoir. While the UNISIM-II dataset incorporates expert knowledge and detailed information about oil production dynamics, it lacks certain complexities, such as time-varying confounders (e.g., choke closure processes) or hidden variables, which can significantly increase the difficulty of causal discovery tasks. Finally, we validated our methodology using real-world Brazilian Pre-Salt oil field data. This involved analyzing two real production datasets, enabling us to demonstrate the practical applicability of our causal discovery framework in real-world oil production scenarios.

### 5.1 Linear semi-synthetic dataset

We construct a linear semi-synthetic dataset by combining real-world fluid injection data with a simplified linear model of production. The generative process includes four components: injected fluid, choke status, a latent state, and production rate. Production is computed using linear, non-lagged combinations of these inputs plus noise.

This setup is designed to simulate confounding through choke behavior, allowing for temporary disruptions in injector-producer connectivity. The latent state captures system variability based on past and current conditions. At each time step, the model iterates over injector data, choke, and latent state to compute production, enabling simulation of diverse operational scenarios, including production halts.

In our application, we simulated 300 independent datasets with a random connection rate of 50%. Our framework was trained using either PCMCI or DYNOTEARS as the causal discovery methods, followed by our proposed classifier framework trained with our causal feature representation. We defined a connection between the injector and producer when the estimated connection probability exceeded a threshold of 0.5.

From Table 2, we draw several insights about the performance of our model across supervised, unsupervised, and hybrid settings. We benchmark it against established unsupervised causal discovery methods for time series, including PCMCI, DYNOTEARS, and the Aleph model for oil fields. As outlined in Section 4, our approach integrates seamlessly with these baselines.

**Table 2:** Performance comparison of accuracy across different methods using semi-synthetic data.

**Method**	**Supervised (1% label)**	**Supervised (5% labels)**	**Supervised (10% labels)**	**Unsupervised**
PCMCI + causal feature (ours)	0.506	0.973	1.00	0.506 (PCMCI)
DYNOTEARS + causal feature (ours)	0.913	0.963	1.00	0.913 (DYNOTEARS)
Causal feature (ours)	0.506	0.903	1.00	-
Aleph (Castro et al., 2023)	-	-	-	0.51

The Unsupervised approach relied solely on the causal discovery algorithm, whereas our method first employs causal discovery and then our classifier model with the features to estimate the probability of connection.

To assess performance under data scarcity, we run experiments using 1%, 5%, and 10% of labeled data, leveraging the known injector-producer relationships as ground truth. With only 1% labels, results align closely with unsupervised methods, indicating early-stage performance is driven largely by unsupervised insights.

As label availability increases (5% and 10%), we observe a clear synergy between supervised and unsupervised components, resulting in improved accuracy beyond what either achieves alone. This highlights the model's ability to enhance causal inference through minimal supervision.

Notably, the classifier alone (without causal discovery algorithms) serves as a performance lower bound. The combination of causal structure and supervised signals consistently yields superior results. Lastly, Aleph performs relatively poorly in this setup, achieving only 51%. After validating the conceptual soundness of our framework on linear data, we next assess its scalability and realism using the UNISIM-II dataset, a more complex synthetic benchmark that captures reservoir heterogeneity and multiphase injection effects.

### 5.2 Synthetic oil production field

We evaluated our algorithms on the UNISIM-II-M-CO dataset, a synthetic benchmark simulating a typical Pre-Salt carbonate reservoir in Brazil (Correia et al., 2015). The UNISIM-II dataset simulates a carbonate reservoir featuring ten producer wells (PRK014, PRK028, PRK045, PRK052, PRK060, PRK061, PRK083, PRK084, and PRK085) and eight injector wells (IRK004, IRK028, IRK029, IRK036, IRK049, IRK050, IRK015, and IRK063), where the initial letter of each well's name indicates its type (P-Producer, I-Injector).

Our analysis focused on Daily Production Oil Rate (DPO) using historical and injection rate data, distinguishing between water and gas injections due to their distinct flow behaviors. Thus, water and gas injectors were treated separately to better reflect reservoir dynamics.

Results were validated against tracer data, which serve as ground truth by confirming injector-producer connectivity through detected chemical compounds. While the absence of tracer signals does not confirm disconnection, our causal methods showed strong alignment with tracer-confirmed links, demonstrating their effectiveness in capturing well interdependencies over time.

**Figure 5:**
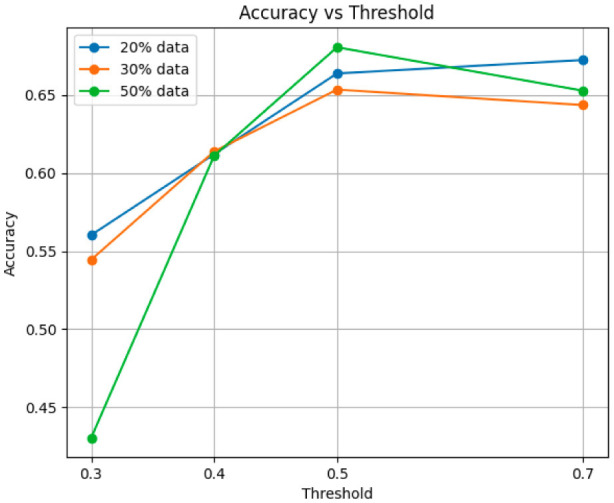
Accuracy metric for training data sizes of 20%, 30%, and 50%.

**Figure 6:**
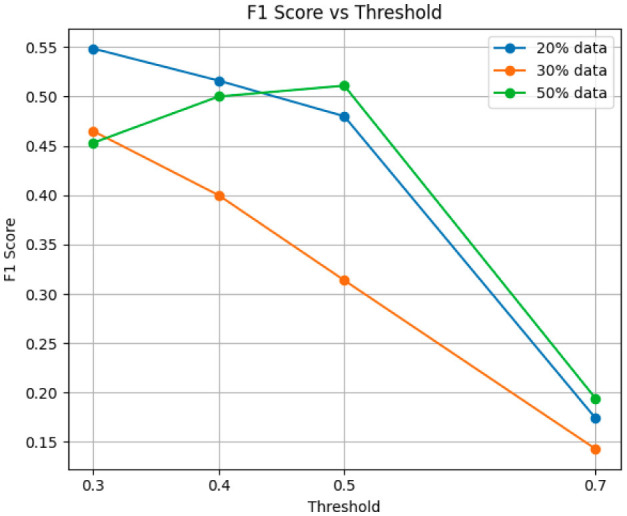
F1 score for training data sizes of 20%, 30%, and 50%.

We begin our evaluation by analyzing the performance of our causal-driven representation learning methods across different thresholds and training sizes, using the validation set for assessment. Figures 5, 6 reports accuracy and F1 score, respectively. While accuracy captures overall correctness, it can be misleading for imbalanced data; the F1 score balances precision and recall, offering a more robust measure. Experiments were conducted with training sizes of 20%, 30%, and 50%, using a fixed seed for comparison.

Table 3 summarizes results for three approaches: (i) our Causal Feature Model, (ii) PCMCI followed by our model, and (iii) DYNOTEARS followed by our model. Here, we validate using the full dataset to reflect real-world oil field application.

**Table 3:** Model performance results in UNISIM-II dataset.

**Model**	**Train size**	**Accuracy (μ ± σ)**	**Precision (μ ± σ)**	**Recall (μ ± σ)**	**F1 score (μ ± σ)**
Causal feature model (our)	0.1	0.60 ± 0.08	0.47 ± 0.07	0.48 ± 0.22	0.44 ± 0.12
	0.2	0.64 ± 0.05	0.52 ± 0.06	0.55 ± 0.16	0.52 ± 0.08
	0.5	0.77 ± 0.02	0.67 ± 0.06	0.71 ± 0.09	0.68 ± 0.03
PCMCI + causal feature model (our)	0.1	0.61 ± 0.03	0.44 ± 0.24	0.17 ± 0.14	0.20 ± 0.15
	0.2	0.62 ± 0.04	0.55 ± 0.21	0.20 ± 0.12	0.25 ± 0.13
	0.5	0.69 ± 0.02	0.71 ± 0.09	0.27 ± 0.05	0.38 ± 0.06
DYNOTEARS + causal feature model (our)	0.1	0.61 ± 0.05	0.52 ± 0.13	0.51 ± 0.27	0.44 ± 0.16
	0.2	0.67 ± 0.04	0.56 ± 0.06	0.58 ± 0.15	0.55 ± 0.06
	0.5	**0.80** **±0.03**	**0.72** **±0.06**	**0.74** **±0.09**	**0.72** **±0.03**

Bold values represent the best performance of our framework.

To assess robustness, we repeated experiments 20 times with different seeds. Table 3 reports accuracy and F1 score for training sizes of 10%, 20%, and 50%. As shown in Figures 5, 6, we selected a classification threshold of 0.4 for balanced performance, based on prior visual analysis to ensure a trade-off between sensitivity and specificity.

From our experiments, and as expected, we observed that the models generally exhibit improved accuracy as the training size increases. This trend suggests that our model performs better in real-world scenarios as we get more labeled data or knowledge about the field. We highlight that in this case, the best performance across all metrics is achieved when identifying potential connections using DYNOTEARS and then applying our causal feature model to refine and learn the connections. However, we assert that the causal feature model itself exhibits good performance, comparable to the optimally tuned configuration.

Of particular significance is the observation that our causal-driven feature model exhibits enhanced performance compared to the PCMCI and DYNOTEARS algorithms. The result is consistent even when employing the most limited training dataset of 10% of the label data provided, which is a good indicator for the usability in real-world data. Having verified our method under controlled and synthetic conditions, we now test its robustness in the most challenging setting—real Brazilian Pre-Salt oil field data.

### 5.3 Real oil production field

Previous approaches for the Brazilian Pre-Salt field often require detailed knowledge of complex reservoir characteristics, making testing and validation in dynamic environments challenging (Castro et al., 2023). In contrast, our approach employs data-driven methods, which alleviate the in-depth understanding of the underlying physics of the oil field, enabling more flexible and scalable solutions for production optimization, reservoir management, and overall field performance—even in heterogeneous settings like the Pre-Salt.

We tested our methods on two real/private datasets from the Brazilian Pre-Salt, a major offshore reserve located around 3,000 meters deep. Known for its thick, high-quality oil-bearing formations beneath a massive salt layer, the Pre-Salt poses several challenges: complex geology, offshore distance, variable CO_2_ content, water and reservoir depth, salt formation intricacies, and flow assurance issues.

We emphasize that while our validation focused on tracer-confirmed connections, the predicted connectivity patterns show qualitative alignment with the geological understanding of the reservoir.

#### 5.3.1 Pre-Salt field 1

The Pre-Salt field comprises 16 producers and 16 injectors, divided into high and low regions based on well locations. We applied our framework to one year of time series data, segmented into scenario windows to ensure wells remained active and roles consistent within each window. Producer variables included oil production, choke pressure, and injector water/gas rates.

**Figure 7:**
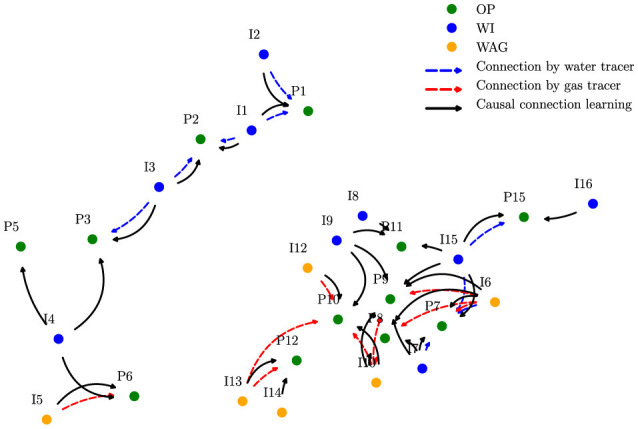
Connectivity map based on causality analysis applied to the Pre-Salt field 1 oil production. The location of each well is shown, and so are established connections based on our method and tracer data.

A connectivity map (Figure 7) of the field illustrates the relationships among various Oil Production (OP), Water Alternating Gas (WAG), and Water Injection (WI) wells, highlighting the interwell connections confirmed by water or gas tracers alongside those detected through our causal methods. In this experiment, we showcase the results when using only five tracers for training instead of the 10 available. With this approach of limiting the known information, we aimed to evaluate whether our model is able to detect the true connectivity, using the remaining tracers as test labels. The detected and confirmed connections identified by our analysis agree strongly; only the connection between injector 24D and producer 681 is missed. This demonstrates the model's ability to generalize under data scarcity.

Notably, training and evaluating 256 pairwise connections took just 38 seconds, emphasizing the scalability and efficiency of our approach for real-world oil field applications.

#### 5.3.2 Pre-Salt field 2

The second Pre-Salt field includes 9 producers and 8 injectors, divided into “low” and “high” regions based on geography. Due to its recency, only contextual data for the low region is available, limiting full-field analysis and posing challenges for connectivity inference. In this field only 3 tracers is available, all presented in the low-region.

Compared to Field 1, this field shows greater variability in well operation (open/closed), making modeling more difficult. To address this, we defined two stable scenario windows (2017–2018 and 2020–2021), each with fixed well states, and applied our framework to one year of data. Producer features included oil production, choke pressure, and injection rates.

**Figure 8:**
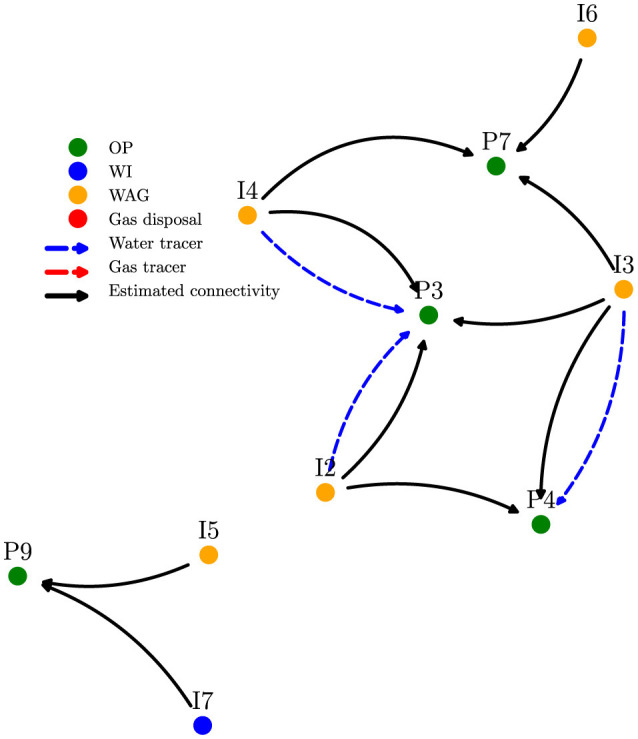
Connectivity map based on causality analysis applied to the low region of the Pre-Salt field. The location of each well is shown, and so are established connections based on our method and tracer data.

**Figure 9:**
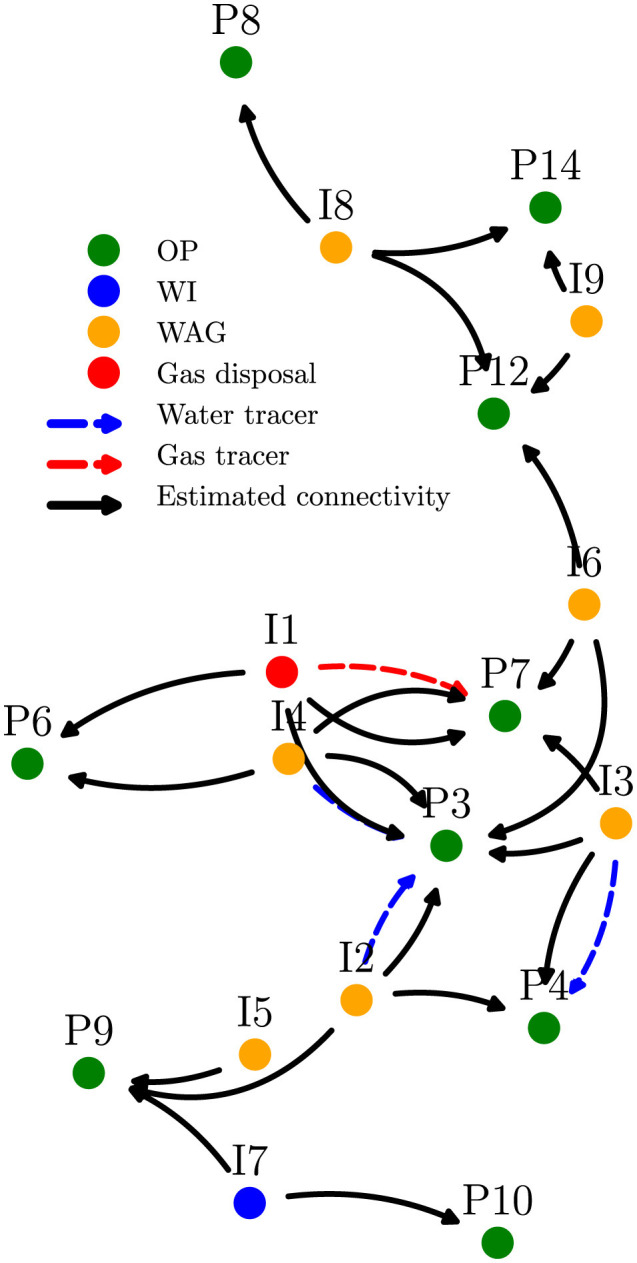
Connectivity map based on causality analysis applied to the complete Pre-Salt field two oil production in Scenario 2. We highlight that all contextual information comes from the low region.

Figure 8 presents results for the low region; Figure 9 shows the inferred connectivity in the full field, despite lacking contextual data in the high region. Training used tracer data only from the low region. The model successfully generalized the learned patterns, accurately predicting connectivity under limited supervision.

## 6 Conclusion and future works

This paper addresses the challenge of inferring connectivity in oil fields using a causal representation learning approach. By formulating the problem through a structured lens, we propose a method that mitigates biases via balanced pairwise representations, enabling robust estimation of injector–producer connections, even with limited data. We validated our approach through experiments on synthetic and semi-synthetic datasets, confirming the generalizability of our model across both controlled conditions and partially real-world scenarios. Furthermore, we extended our analysis to the Brazilian Pre-Salt fields, a geologically complex environment. Results show our method can overcome common challenges in oil fields for connectivity, such as missing pressure data, human intervention uncertainty, and noisy production rates, and they were consistent across various scenarios. This is advantageous in the oil field, where such contextual information is often expensive and time-consuming, and experiments are nearly prohibited. These factors seem a promising finding over other causal approaches mentioned earlier (see Section 2) that often face challenges when applied to real-world oil field scenarios, and in turn offering a scalable and performative solution that might enhance decision-making within complex dynamic fields.

In the real-field validation, we adopted a conservative evaluation strategy restricted to well pairs with confirmed tracer-based connectivity, rather than attempting to infer geological consistency directly, i.e., level of connectivity low or high diffusion. This choice ensures reliability in positive detections but limits the current framework's ability to assess the alignment between predicted connectivity and detailed geological structures, a direction future research can explore further.

Despite these promising results, some limitations remain. In particular, the method's performance may depend on the choice of the time-window length used to construct the pairwise scenarios. A window that is too short may fail to capture meaningful interactions, while one that is too long may dilute transient connectivity patterns. A systematic sensitivity analysis regarding this temporal parameter is an important direction for future work. Another limitation concerns the method's robustness under highly dynamic operational conditions, such as frequent well shut-ins or abrupt changes in injection strategies, that can alter system dynamics faster than the comparative features can adapt. Extending the approach to handle these non-stationary regimes represents an interesting challenge.

We argue that, beyond oil field applications, our formulation of connectivity estimation through causal feature learning opens avenues for similar analyses in other domains where entities interact through covariant measurable signals. Examples include detecting causal links between climate variables, identifying influence networks in energy systems, or uncovering interdependencies in social processes. Even in the presence of partial confounding, our empirical results suggest that comparative causal features provide a simple yet powerful framework for identifying meaningful dependencies between two functional entities.

Future work will explore end-to-end deep learning for causal representation learning, leveraging the causal feature approaches to create more expressive high-level models and unsupervised clustering to improve structure discovery further. Ultimately, identifying the connectivity map sets the stage for downstream causal inference, estimating the impact of injectors on production, supporting more accurate, cost-effective reservoir management, and decision-making.

## Data Availability

The original contributions presented in the study are included in the article/Supplementary material, further inquiries can be directed to the corresponding author.
