# Supplementary material for: Causality-driven feature representation for connectivity prediction
Source: Front Artif Intell. 2026 Jan 15;8:1686750. doi: 10.3389/frai.2025.1686750 (PMC12852402; doi:10.3389/frai.2025.1686750)
Supplement: Supplementary file 1 [file Data_Sheet_1.pdf]

Supplementary Material

1 TRAINING SETUP

In this section, we delineate our training setup for identifying connections between injector-producer pairs using a structured causal discovery framework and causal features for the classifier model.

1.1 The causal discovery algorithms

Our framework starts by constructing a mutually independent pairwise list of all injector-producer combinations, operating under the assumption that the connections between these pairs are independent of one another. This list serves as the foundation for applying two distinct causal discovery algorithms, the PCMCI and the DYNOTEARS, which are employed to refine the list by filtering out unlikely connections. Specifically, in our case, when both algorithms indicate that no connection exists between a pair, that pair is subsequently removed from consideration.

The use of causal discovery algorithms before the classifier model is intended to have a double evaluation on the negative connection, therefore, as an initial approach, we aim to impose minimal restrictions during this filtering process. Subsequently, recognizing the importance of hyperparameter configuration in causal discovery, we set the alpha hyperparameters to 0.3 for PCMCI and 0.05 for DYNOTEARS. These hyperparameters, in practice, are less restricted. This strategy is designed to ensure that only the most probable non-connected pairs are filtered out, thereby preserving as many relevant connections as possible for further analysis.

1.2 Training setup and the bootstrapping strategy

Following this initial filtering, we create a causal pairwise feature representation, as explained in Section Methodology. Utilizing this representation in conjunction with our limited context data as positive samples, we proceed to train our classifier model. We opted for a random forest model due to its numerous advantages, including its inherent ability to handle high-dimensional data and its robustness against overfitting, along with the short time it takes to train, which is beneficial for scalability in real-world scenarios. Additionally, after training, random forest models enable the estimation of the likelihood of a positive label for test data, which is instrumental for our analysis.

Given the absence of readily available negative labels, indicating that certain pairs are not connected, we employ expert knowledge regarding the distances between the injector and producer location to facilitate the creation of negative labels. Specifically, pairs with considerable distances are deemed very likely to be unconnected. This approach generated a substantial number of negative labels as compared to positive ones. The greater quantity of negative labels biased our model to predict low scores when performing classification analysis.

To address the potential for overfitting, given this imbalance, we apply a bootstrapping approach. Bootstrapping involves sampling with replacement from our dataset to create multiple training subsets, thereby enhancing the robustness and generalizability of the model by exposing it to various scenarios (i.e., note the positive label several times within different configurations) and obtaining different estimations of the connections. This approach allow us to have uncertainty in the estimation and therefore be more confident in the decision about the connectivity of injector and producer. In our framework, we apply the bootstrapping resampling technique 100 times by generating datasets through sampling with replacement.

Each trained model produces a probability estimate for each connection in the dataset. We then compute the final probability of each connection by averaging these estimates across all models. Given it scalability and easy-to-use approach, we use a random forest model of the *scikit learn* package with standard configuration.

1.3 Threshold hyperparameter control

Upon completion of the training phase, we apply a threshold on the connection predictions generated by the trained model. This threshold serves to filter out lower-scoring pairs, retaining only those that exceed the designated threshold, which the model considers to be connected. This systematic approach enables us to effectively identify and validate potential connections within the framework. In practice, the operators can change the threshold to evaluate the best connection configuration the model provides. This controllability is beneficial for insights and decision-making in oil field, enhancing the reliability of the findings.

2 UNISIM-II RESULTS

We evaluated the performance of the unsupervised PCMCI and DYNOTEARS algorithms on the UNISIM-II dataset. Table S1 summarizes the results obtained using a lag max of 10 steps in time for both algorithms, and alpha values of 0.2 and 0.1 for PCMCI and DYNOTEARS, respectively.

Table S1. Model Performance Results in UNISIM-II Dataset

Model	Accuracy	Precision	Recall	F1 Score
**PCMCI**	0.55	0.31	0.21	0.25
**DYNOTEARS**	0.41	0.37	0.94	0.54

As shown in Table S1, the two algorithms exhibit contrasting performance characteristics. PCMCI achieves a moderate accuracy of 0.55, but its precision of 0.31 and recall of 0.21 are low, resulting in a low F1-score of 0.25. This suggests that PCMCI struggles to both accurately identify true relationships and capture a significant portion of all existing relationships.

DYNOTEARS, on the other hand, shows a lower accuracy of 0.41 but a much higher recall of 0.94. This indicates that DYNOTEARS is very effective at identifying nearly all existing relationships. However, this comes at the cost of lower precision, 0.37, meaning it also identifies many false relationships. The F1-score for DYNOTEARS of 0.54 is significantly higher than PCMCI, reflecting its ability to better balance precision and recall.

3 UNISIM EVALUATION METRICS

To gain a deeper understanding of the threshold hyperparameters and the performance of the model on the UNISIM-II benchmark, we conduct further analyses that include the evaluation of recall and precision. Recall measures the ability of the model to identify positive instances, while precision assesses the accuracy of those positive predictions. Both metrics are essential for evaluating the trade-offs between sensitivity and specificity in classification tasks.

The Figure S1 and Figures S2 illustrates the performance curves for recall and precision as the threshold varies across different proportions of trainable labels (20%, 30%, and 50%) in the validation set. As shown, recall decreases as the threshold increases, indicating a trade-off between detecting true positives and the

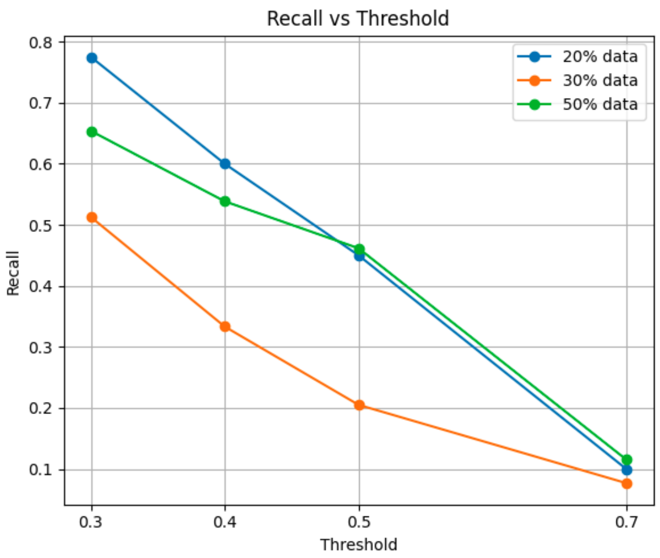

Figure S1. Recall as a function of varying thresholds. Evaluation on the validation set.

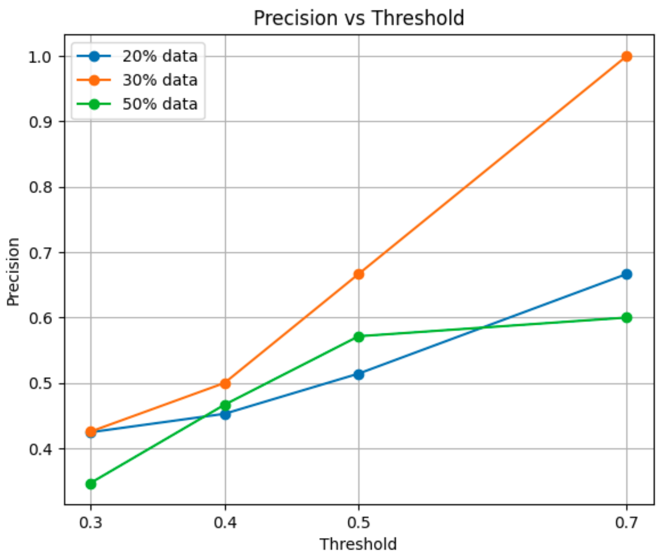

Figure S2. Precision as a function of varying thresholds. Evaluation on the validation set.

criteria needed to classify an instance as positive. Conversely, precision demonstrates a monotonic increase with higher thresholds, revealing that as the threshold rises, the proportion of true positive predictions relative to all positive predictions improves.

Finally, Figure S3 and Figure S4 illustrate the roc curve and the precision vs recall curve using different proportion of trainable labels. As show, mainly in the roc curve the approach performs relatively well with few labeled data and tends to increase the performance as we get more data.

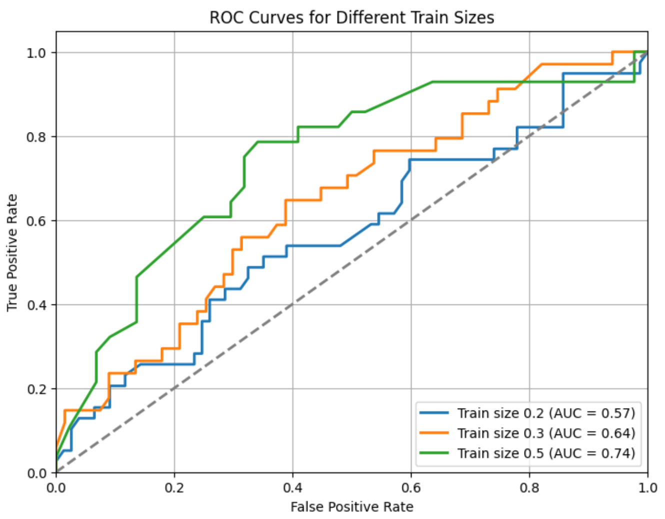

Figure S3. ROC curve as a function of training data size.

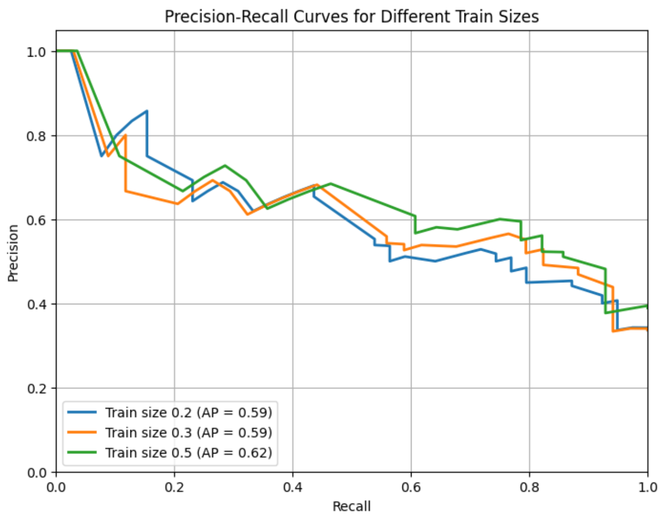

Figure S4. Precision vs. Recall curve as a function of training data size.

4 SEMI-SYNTHETIC DATA GENERATION

4.1 DAG of Semi-synthetic dataset

In line with the principles of the Structural Causal Model, we constructed our semi-synthetic dataset based on a directed acyclic graph and its corresponding structural functions. The figure below illustrates the graphical representation of the data generating process.

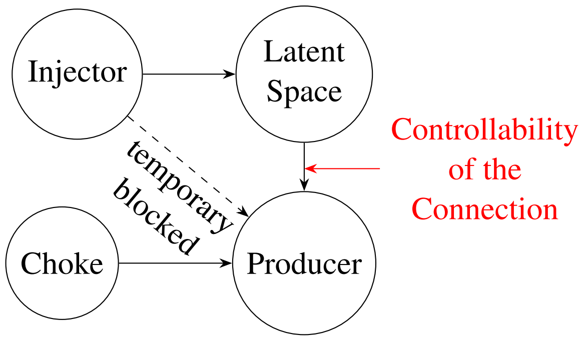

The connectivity between the Producer and Injector is effectively illustrated by the solid arrow pointing from the Latent Space to the Producer. The label ”Controllability of the Connection” indicates the point at which we can enable or disable this connection, depending on whether the Injector is linked to the Producer.

The dashed line between the Injector and Producer represents a temporarily blocked connection forced by the Choker. This indicates that while the Injector typically sends resources directly to the Producer, this flow can be temporarily interrupted by the Choker. When the Choker is activated, it blocks connectivity, preventing the Producer from receiving input directly from the Injector. Even though the Injector and Producer are connected, temporarily blocked connections are allowed to simulate real-world Choke's behavior.

4.2 Functions of Semi-synthetic dataset

We develop a comprehensive methodology to generate synthetic data for four variables: A, a pressure of the choke, a latent state, and B. This process integrates mathematical modeling with stochastic components, mimicking real-world phenomena and providing a basis for causal analysis. It is worth noting that the general idea is to simulate the behavior of the choke (i.e., close the connectivity temporarily) and additionally with the presence of a latent variable.

The function defines three critical inner functions:

1. **Function** $f(a_t)$: Generates values for variable A. Although $f(a_t)$ could represent any autoregression function, possibly with noise, in our case, the variable A comes from a real fluid injected.
2. **Function** $g(U_c, \texttt{Choke}_t)$: This function is used to model the choke behavior, with noisy value U_c:

$$U_c = \begin{cases} 0 & \text{with probability } 0.4 \cdot \text{Simulate closed choke} \\ 0.4 + 0.5 \cdot \text{rand}(U_c) & \text{Simulate value higher than 0.4 (i.e., as usually find in real values)} \end{cases}$$

   This introduces variability in the confounder based on its previous state, ensuring it retains some stochastic characteristics while allowing for possible reset to zero under certain conditions.

The core of the synthetic data generation occurs within a loop that iterates through each time step t:

- **Noise Initialization:** At each step, U_a is updated using the previous injector's fluid point data, while U_c is sampled from a predefined range, and U_b is drawn from a uniform distribution.
- **Generate** A: $A(t) = f(U_a)$
- **Generate Confounder**: $\texttt{Choke} = g(h(U_c), \texttt{Choke}_{t-1})$

- **Latent State Determination**:

$$\texttt{State}(t) = \begin{cases} 0 & \text{if } \texttt{Choke}_t = 0 \\ \alpha \cdot A(t) + \text{noise} & \text{otherwise} \end{cases}$$

- **Output Generation** B:

$$B(t) = \begin{cases} \alpha \cdot \sin(U_a) \cdot A(t) + \beta \cdot \sin(U_c) \cdot \texttt{State}(t) + \text{noise} & \text{if connection is } \texttt{True} \\ \beta \cdot \texttt{Choke}_t + \text{noise} & \text{if connection is } \texttt{False} \end{cases}$$

$$B(t) = \begin{cases} 0 & \text{if } \texttt{Choke} \text{ is closed independently of the connectivity} \end{cases}$$

By utilizing this mathematical framework, we create a synthetically generated dataset that mirrors the complexities of real-world interactions within a system governed by latent influences and factors related to the chokes, which brings additional complexity to the task of connectivity discovery. This systematic approach facilitates exploring the intricate relationships between injectors and producers, significantly enhancing our capacity to analyze connectivity in subsurface flow systems.

5 MATHEMATICAL FORMULATIONS OF THE REPRESENTATION

Below, we provide formal definitions for the connectivity representation introduced in Section **??**.

1. **Maximum Correlation**: The maximum correlation measures the linear dependence between injector and producer signals. High values suggest strong synchronization. The linear correlation coefficient ρ between injector $I(t)$ and producer $P(t)$ is given by:

$$\rho = \frac{\text{Cov}(I, P)}{\sigma_I \sigma_P}$$

   where Cov is covariance and σ denotes standard deviation.

2. **Granger Causality**: The method tests if injector data improves producer predictions. A causal relationship implies directional influence.

   a. **Restricted model** (null hypothesis):

$$P(t) = \sum_{k=1}^{K} \alpha_k P(t-k) + \epsilon_t \tag{S1}$$

   b. **Unrestricted model** (alternative hypothesis):

$$P(t) = \sum_{k=1}^{K} \alpha_k P(t-k) + \sum_{k=1}^{K} \beta_k I(t-k) + \epsilon'_t \tag{S2}$$

   The F-statistic tests the null hypothesis $H_0 : \beta_k = 0$ for all $k \in \{1, ..., K\}$. If β_k is not 0 we say that injector I granger-causes the producer P.

3. **Mutual Information (MI)**: The MI quantifies the non-linear shared information. Quantifies the reduction in uncertainty about production given injection data. In addition, MI measures both linear

and non-linear dependencies and it is robust to non-Gaussian distributions common in reservoir data. The mathematical equation is given by:

$$\text{MI}(I, P) = \sum_{i,j} p(i,j) \log \frac{p(i,j)}{p(i)p(j)}$$

where $p(i,j)$ is the joint distribution and $p(i)$, $p(j)$ are marginals.

4. **Power Spectral Density (PSD) Correlation**: The Power Spectral Density Correlation identifies synchronized frequency components between injector and producer signals. The PSD $S_{II}(f)$ of $I(t)$ and cross-PSD $S_{IP}(f)$ are computed via Fourier transforms. Their coherence is:

$$C_{IP}(f) = \frac{|S_{IP}(f)|^2}{S_{II}(f)S_{PP}(f)}$$

The key concepts is to identify the peaks at specific frequencies indicate periodic connectivity patterns, leverage the detection delayed responses through phase analysis and be a complements to time-domain metrics.

5. **Conditional Mutual Information (CMI)**: The Conditional Mutual Information measures the unique information between injector and producer after accounting for confounding factors, choke pressure in our case. The idea is that the difference isolates the confounder-free relationship CMI between I and P given choke pressure C:

$$\text{CMI}(I; P|C) = \text{MI}(I, P) - \text{MI}(I, P|C)$$

6. **Distance Metric**: The Euclidean distance d between injector and producer coordinates (x_I, y_I) and (x_P, y_P):

$$d = \sqrt{(x_I - x_P)^2 + (y_I - y_P)^2}$$
